# Genomic, metabolic and phenotypic variability shapes ecological differentiation and intraspecies interactions of *Alteromonas macleodii*

**DOI:** 10.1038/s41598-020-57526-5

**Published:** 2020-01-21

**Authors:** Hanna Koch, Nora Germscheid, Heike M. Freese, Beatriz Noriega-Ortega, Dominik Lücking, Martine Berger, Galaxy Qiu, Ezequiel M. Marzinelli, Alexandra H. Campbell, Peter D. Steinberg, Jörg Overmann, Thorsten Dittmar, Meinhard Simon, Matthias Wietz

**Affiliations:** 1 Institute for Chemistry and Biology of the Marine Environment, University of Oldenburg, Oldenburg, Germany; 2 Leibniz Institute DSMZ - German Collection of Microorganisms and Cell Cultures, Braunschweig, Germany; 3 ICBM-MPI Bridging Group for Marine Geochemistry, University of Oldenburg, Oldenburg, Germany; 4 Centre for Marine Science and Innovation, University of New South Wales, Kensington, Australia; 5 Singapore Centre for Environmental Life Sciences Engineering, Nanyang Technological University, Singapore, Singapore; 6 Sydney Institute of Marine Science, Mosman, Australia; 7 Braunschweig University of Technology, Braunschweig, Germany; 8 Present Address: Radboud University Nijmegen, Nijmegen, The Netherlands; 9 Present Address: Leibniz Institute of Freshwater Ecology and Inland Fisheries, Berlin, Germany; 10 Present Address: Western Sydney University, Hawkesbury, Australia; 11 Present Address: University of Sydney, Camperdown, Australia; 12 Present Address: University of Sunshine Coast, Sunshine Coast, Australia; 13 Present Address: Alfred Wegener Institute Helmholtz Centre for Polar and Marine Research, Bremerhaven, Germany

**Keywords:** Marine microbiology, Water microbiology

## Abstract

Ecological differentiation between strains of bacterial species is shaped by genomic and metabolic variability. However, connecting genotypes to ecological niches remains a major challenge. Here, we linked bacterial geno- and phenotypes by contextualizing pangenomic, exometabolomic and physiological evidence in twelve strains of the marine bacterium *Alteromonas macleodii*, illuminating adaptive strategies of carbon metabolism, microbial interactions, cellular communication and iron acquisition. In *A. macleodii* strain MIT1002, secretion of amino acids and the unique capacity for phenol degradation may promote associations with *Prochlorococcus* cyanobacteria. Strain 83-1 and three novel Pacific isolates, featuring clonal genomes despite originating from distant locations, have profound abilities for algal polysaccharide utilization but without detrimental implications for *Ecklonia* macroalgae. Degradation of toluene and xylene, mediated via a plasmid syntenic to terrestrial *Pseudomonas*, was unique to strain EZ55. Benzoate degradation by strain EC673 related to a chromosomal gene cluster shared with the plasmid of *A. mediterranea* EC615, underlining that mobile genetic elements drive adaptations. Furthermore, we revealed strain-specific production of siderophores and homoserine lactones, with implications for nutrient acquisition and cellular communication. Phenotypic variability corresponded to different competitiveness in co-culture and geographic distribution, indicating linkages between intraspecific diversity, microbial interactions and biogeography. The finding of “ecological microdiversity” helps understanding the widespread occurrence of *A. macleodii* and contributes to the interpretation of bacterial niche specialization, population ecology and biogeochemical roles.

## Introduction

Metabolic variability is a major driver of ecological differentiation within bacterial taxa, shaping adaptive strategies and hence the niche space of related strains^1^. With the increasing number of sequenced genomes, substantial functional diversity is being discovered among closely related strains^2^, with implications for bacterial species concepts^3^. This diversity can be investigated by interrogating the pangenome of a taxonomic group (i.e. their entire repertoire of core and variable genes) for genotypic variants with ecological implications^4^. Ecological differentiation within a taxon mainly relates to two flexible genomic categories: the accessory genome (shared by several strains) and the unique genome (restricted to individual strains). This variable repertoire is often encoded in genomic islands, hotspots of genetic exchange^5^ known to influence niche specialization in cyanobacteria, actinobacteria and roseobacters^6–8^. Flexible genomic islands, located at equivalent loci in different strains of the same taxon, can provide or replace genetic information and are important factors for intraspecific heterogeneity^9,10^, for instance governing carbon utilization, siderophore production and pilus assembly^11^. These adaptive-evolutionary processes are often amplified by plasmids and other mobile genetic elements, driving horizontal gene transfer (HGT) and diversification on short time scales^12–14^. Bacterial adaptations can also relate to single-nucleotide exchanges via homologous recombination or mutations^15,16^.

Current approaches to species delineation, such as 16S rRNA or core-genome phylogenies, do not always reflect the diversity of strain-specific ecological strategies. For instance, the analysis of ~400 *Vibrio cholerae* strains has revealed distinct intraspecific variability in genes mediating bioluminescence and colonization of zooplankton^17^. Closely related vibrios also show substantial divergence in polysaccharide degradation^18^ and particle colonization^19^. Comparable diversity has been observed for biosynthetic capacities within marine *Salinispora* species^20^, with implications for strain-specific competitive abilities^21^. Also the degree of carbohydrate utilization can vary between strains of the same species^22^. Recently, these aspects have been extended to the metapangenomic dimension, revealing linkages of genomic and geographic variability among *Prochlorococcus* strains^23^.

The marine gammaproteobacterium *Alteromonas macleodii* is an excellent model to study the ecological consequences of strain-level variability, as multiple genome-sequenced isolates from diverse habitats and locations are available. The occupation of different niches^24^, varied interactions with other organisms^25–27^ and utilization of diverse substrates^28,29^ suggests the existence of functionally distinct entities within the *A. macleodii* species boundary, despite being >99% identical on 16S rRNA gene level. This notion is supported by the diverse flexible genome and a high degree of genetic exchange between *A. macleodii* and the “sister species” *A. mediterranea*^13,30^. Consequently, genomic islands and mobile genetic elements are major drivers of genetic and metabolic variability within *Alteromonas*, influencing surface-associated vs. free-living lifestyles^31^, exopolysaccharide production^30^, heavy metal resistance^32^ and polysaccharide utilization^33^. Notably, co-occurring *Alteromonas* strains have been postulated to colonize distinct microniches based on specific genomic features^34^ and competitive abilities^35^. For instance, *A. mediterranea* strains differ in motility and glucose utilization, potentially influencing patterns of co-occurrence or mutual exclusion^35^. Despite these ecological implications of genome plasticity, phenotypic and genomic variability have not been comprehensively linked in *Alteromonas* to date, largely because few putative traits have been experimentally verified.

The present study investigated strain-level phenotypic and genomic variability in twelve strains of *A. macleodii* with completely sequenced genomes, including three novel isolates from a Pacific Ocean transect. Supported by exometabolomic evidence and targeted physiological assays, we show how accessory and unique features shape ecological differentiation and result in “microdiversity” of phenotypic traits^1,36^. Co-culturing experiments linked these observations to strain-specific competitiveness, a factor that may influence ecophysiological roles and biogeographic distribution. The finding of diverse metabolic potentials within a narrow taxonomic range, whose members may co-occur or compete depending on prevailing conditions, contributes to the functional interpretation of bacterial species and populations. The shown intraspecific diversity in adaptive strategies helps understanding the widespread occurrence of *A. macleodii* in the oceans, with broader implications for bacterial population ecology and niche specialization.

## Results and Discussion

**Figure 1:**
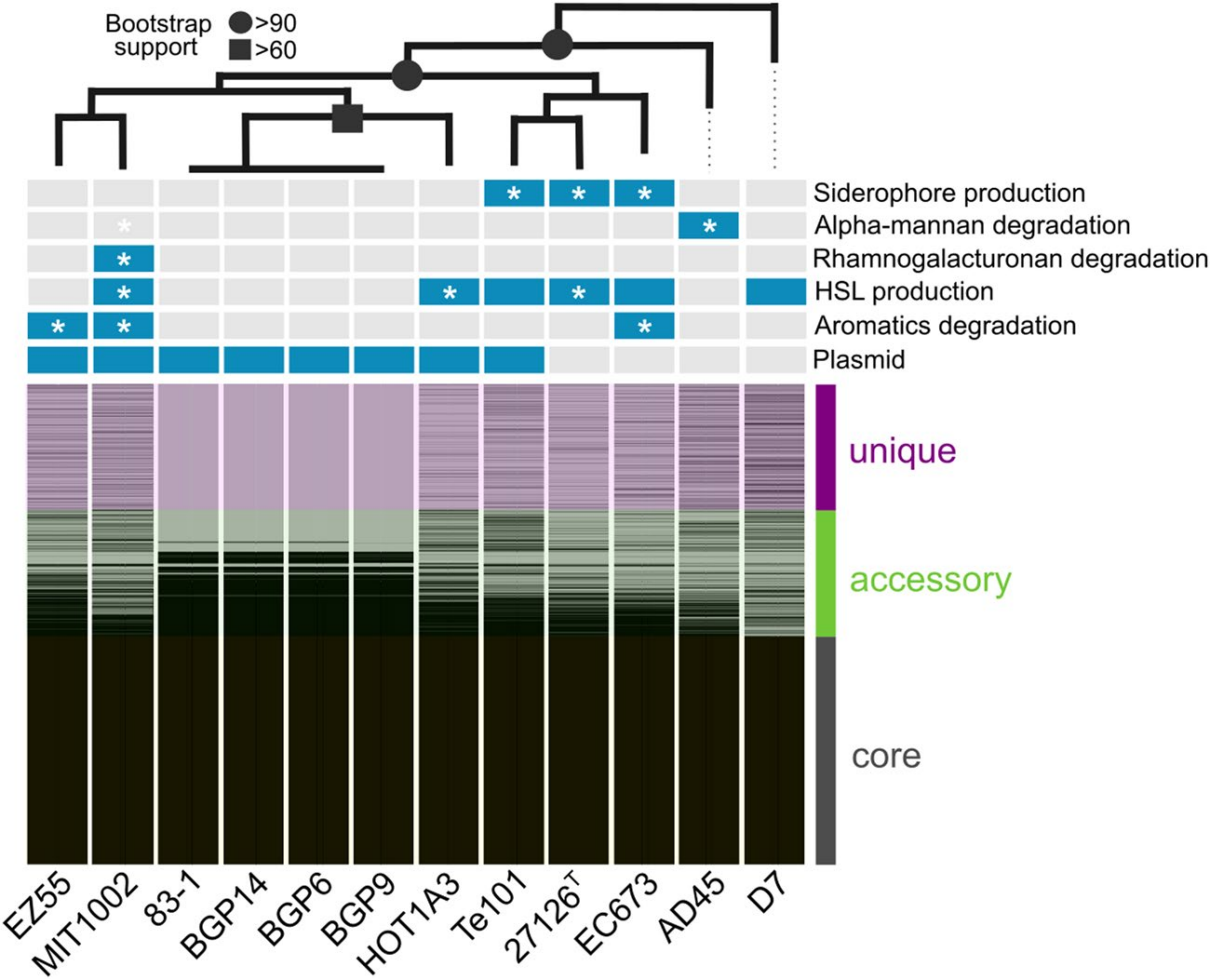
Maximum-likelihood phylogeny and pangenome structure of *Alteromonas macleodii*, showing presence (blue) and absence (gray) of specific genomic features. Phylogenetic analysis was based on 92 single-copy housekeeping genes identified using the UBCG pipeline^119^. Asterisks designate phenotypic features experimentally verified in the present study. Bootstrap support values are indicated by symbols; unlabeled branches have <50% support.

This study combines genomic and phenotypic evidence to illuminate mechanisms of ecological differentiation within *Alteromonas macleodii*, a bacterium with widespread distribution and biogeochemical importance in the oceans^24^. The study focused on twelve *A. macleodii* strains with closed genomes, featuring average nucleotide identities (ANI) of 96.5–99.9% and 16S rRNA gene similarities of >99% (Fig. 1; Table S1). Despite this clear association to a single genospecies^37^, underlined by 3002 core genes, we detected considerable strain-level diversity related to 1662 accessory and 1659 unique gene clusters (Table S2). This is consistent with the pronounced diversity of the flexible genome in *A. macleodii* and the “sister species” *A. mediterranea*, as described previously^30^. Intraspecific differences were highlighted by a diverse pan-exometabolome of 138 core, 1796 accessory and 2096 unique molecular masses secreted during late exponential growth (Table S3). In the following, we contextualize (pan)genomic and phenotypic evidence to characterize how genome plasticity shapes interactions with cyanobacteria and macroalgae, degradation of aromatics and polysaccharides, chemical communication, iron acquisition, and intraspecific competition. These insights expand structural-genomic and evolutionary aspects of the *Alteromonas* pangenome^30,32,34,38,39^ by ecological perspectives on niche specialization, competitive abilities and biogeography.

### Plasmids and genomic rearrangements

As niche specialization is often mediated by mobile genetic elements^40^, we first characterized occurrence and function of plasmids. Eight out of twelve *A. macleodii* strains, including MIT1002 and EZ55 whose genomes were re-sequenced and closed herein, were found to contain a plasmid (Figs. 1, S1). Synteny of plasmids from *A. macleodii* Te101 and *A. mediterranea* DE1 corroborates the role of plasmids for niche specialization within and across species boundaries^13,41^.

**Figure 2:**
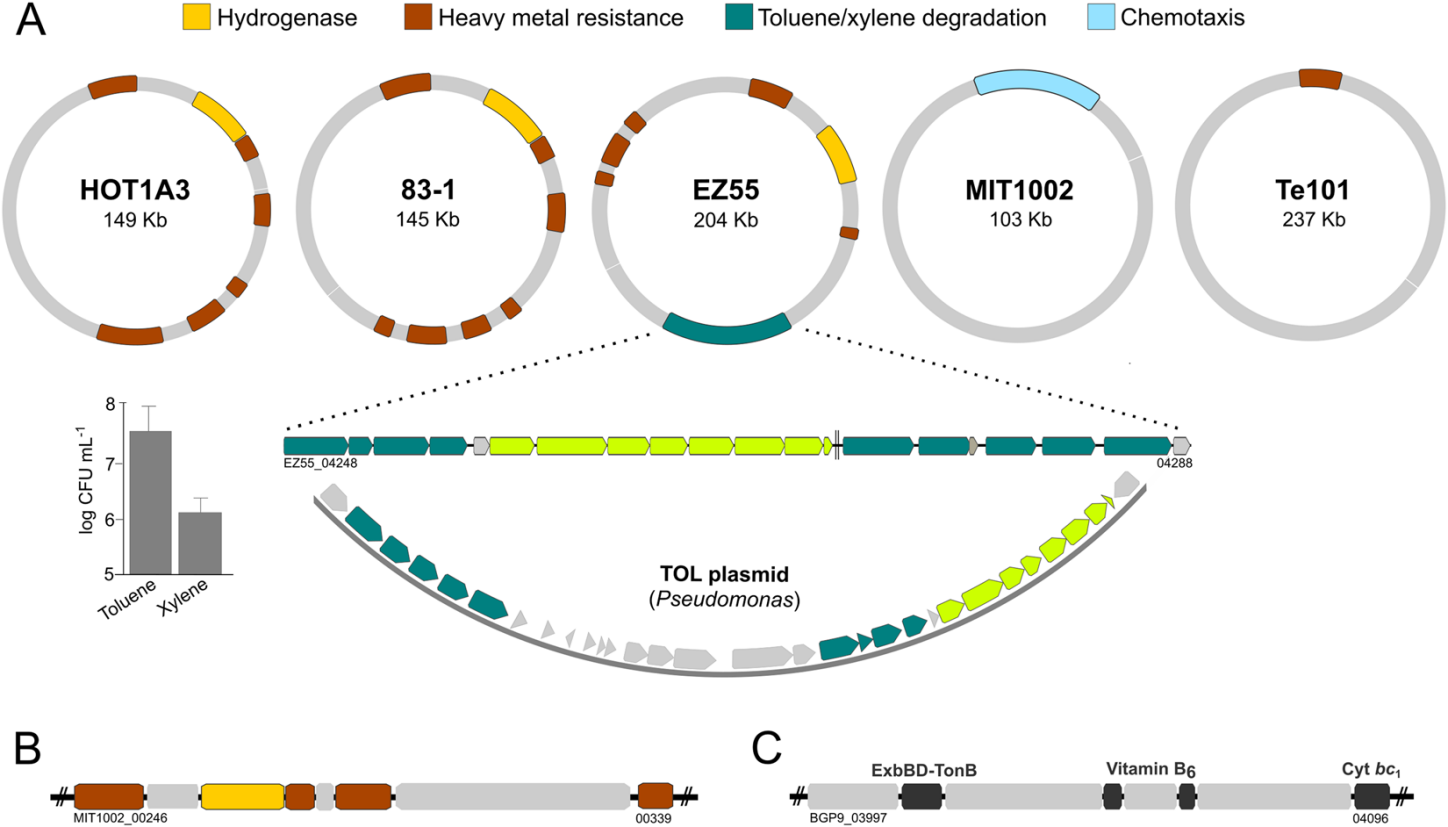
Structural diversity of plasmids in *Alteromonas macleodii*. (**A**) Functionally similar plasmids in strains HOT1A3, 83-1 and EZ55 encoding hydrogenase and heavy metal resistance cassettes, however with different organization. The plasmid of EZ55 furthermore contains a unique insertion syntenic to the *Pseudomonas* TOL plasmid (blue-green: toluene/xylene hydroxylases and transporters; green: catechol meta-cleavage pathway; gray: non-homologous genes) allowing growth with toluene and xylene as sole carbon source (insert). The plasmid of strain Te101 is structurally different and encodes only one resistance cassette. (**A**,**B**) Strain MIT1002 harbors a unique chemotaxis-related plasmid, whereas an 80 Kb region encoding hydrogenase and resistance cassettes has been translocated to the chromosome. (**C**) Strain BGP9 features a chromosome-plasmid translocation of a 90 Kb region harboring a TonB/ExbBD membrane system, a cytochrome *bc*_1_ complex and vitamin B_6_ synthesis genes.

The plasmids of six strains display a similar functional profile, harboring metal resistance and [NiFe] hydrogenase cassettes (Fig. 2A) that have been described in *Alteromonas* before^42,43^ and provide increased resistance compared to strains lacking these cassettes^43^. As homologous cassettes in *A. mediterranea* are encoded in a chromosomal genomic island^30,32,44^, plasmids possibly mediate their transfer between Alteromonadales^13^. Notably, number and arrangement of cassettes differed between strains (Fig. 2A), which may result in varying expression levels and hence different resistance profiles^45^. In strain MIT1002, hydrogenase and resistance cassettes have been inserted into the chromosome, and a unique chemotaxis-related plasmid has been acquired (Fig. 2B). This event may enhance chemosensory abilities and provide a competitive advantage to access nutrient patches^46^.

**Figure 3:**
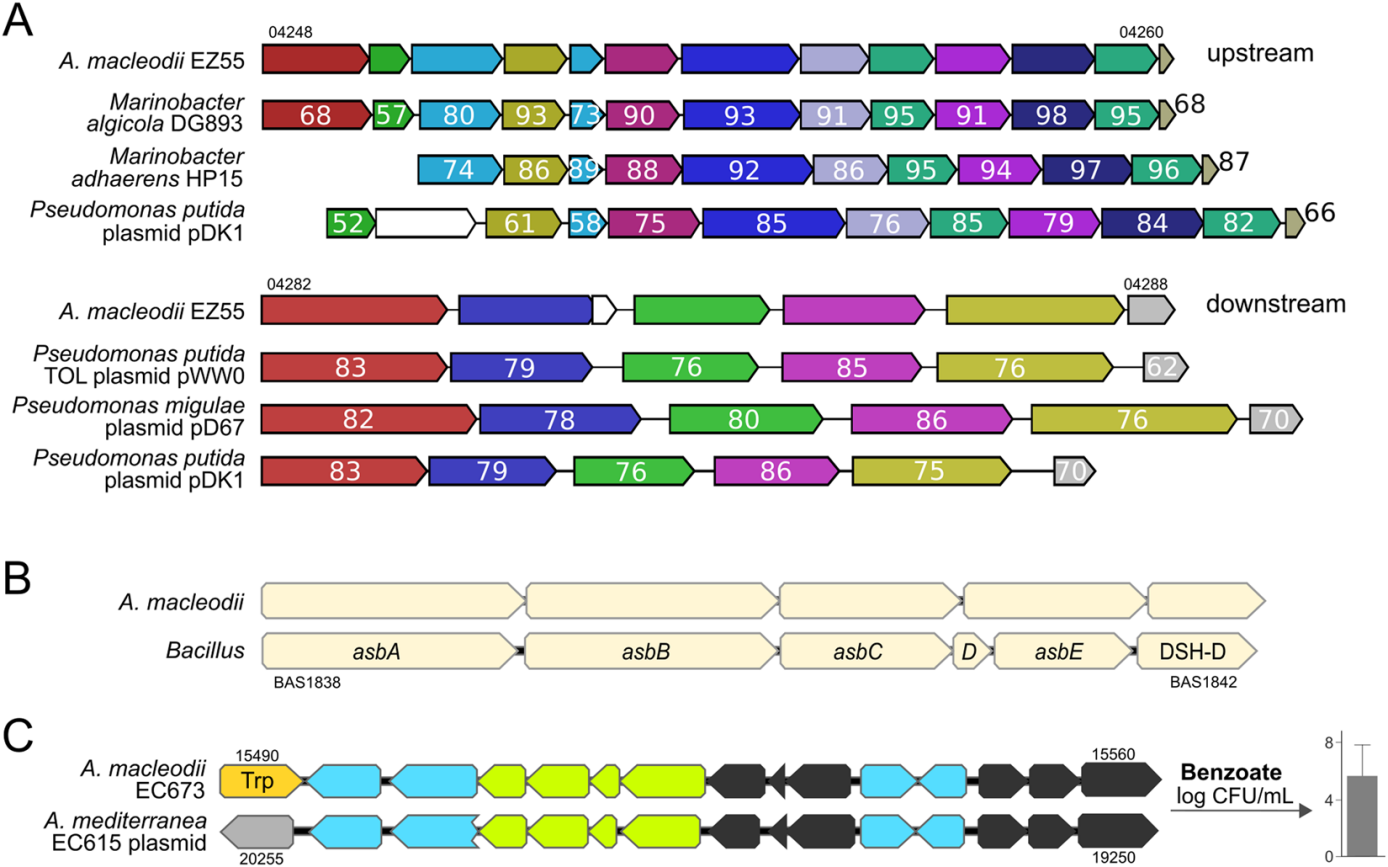
Comparative analysis of selected gene clusters in *Alteromonas macleodii* and other bacteria. (**A**) Gene cluster for toluene/xylene degradation in strain EZ55 plus closest relatives of upstream (locus tags 04248–04260) and downstream (04282–04288) cluster sections. Colors illustrate homologs as determined by MultiGeneBlast, with numbers designating % amino acid similarities. (**B**) Homology of the siderophore-encoding cluster of strains ATCC27126^T^, EC673 and Te101 with the petrobactin operon *asbABCDE* plus adjacent dehydroshikimate dehydratase (DHS-D) from *Bacillus* spp. (**C**) Gene cluster for benzoate degradation in strain EC673, encoding benzoate dioxygenases (green), the catechol ortho-cleavage pathway (black) and transporters/regulators (blue), allowing growth with benzoate as sole carbon source (right insert). A homologous cluster is encoded on the plasmid of *A. mediterranea* EC615. Trp: transposase; gray: non-homologous gene.

The plasmid of strain EZ55 harbors a unique 20 Kb insert, enabling aerobic degradation of the aromatic hydrocarbons toluene and xylene (Figs. 2; S2) as rarely described in marine microbes to date^47,48^. The insert is overall homologous to the TOL plasmid from *Pseudomonas putida* (Fig. 2A), a hydrocarbon-degrading Gammaproteobacterium from soil^49^. However, closer examination using MultiGeneBlast^50^ suggests assembly during separate horizontal transfer events. Specifically, the downstream section (locus tags 04282–04290) has highest similarity to TOL plasmids from *Pseudomonas* strains, with amino acid identities between 70 to 86% (Fig. 3A). In contrast, the upstream section including the catechol meta-cleavage pathway (locus tags 04248–04260) has highest similarity to homologous clusters in *Marinobacter* followed by *Pseudomonas* spp., with amino acid identities between 52 and 98% (Fig. 3A). Considering multiple adjacent transposases and recombinases (locus tags 04244, 04264, 04266, 04267, 04270, 04273, 04279, 04291) and the fact that *Alteromonas*, *Pseudomonas* and *Marinobacter* co-occur during oil spills where toluene and xylene are present^51^, we hypothesize exchange of these clusters at contaminated sites. Alternatively, *Marinobacter* might constitute a “vehicle” between soil and seawater due to its occurrence in saline lakes und intertidal areas^52^ and known acquisition of aromatic-degrading genes from *Pseudomonas*^53^. Considering the common association of *Marinobacter* spp. with phototrophs^54,55^, the cluster might likewise enable degradation of ecologically more relevant aromatics from cyanobacteria, e.g. derivatives of benzoate or cinnamate^56^.

### *Alteromonas* and *Prochlorococcus*

**Figure 4:**
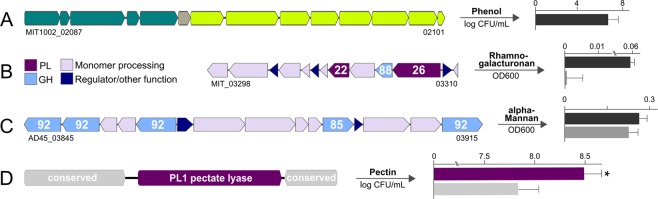
Features of *Alteromonas macleodii* relating to interactions with cyanobacteria and macroalgae. (**A**) Unique gene cluster in strain MIT1002 encoding phenol hydroxylases (blue-green) and the catechol meta-cleavage pathway (green), allowing growth with phenol as sole carbon source. (**B**,**C**) Unique polysaccharide utilization loci in strains MIT1002 and AD45 allowing growth with rhamnogalacturonan and alpha-mannan as sole carbon source (MIT1002: dark gray, AD45: light gray). Numbers designate encoded glycoside hydrolase and polysaccharide lyase families. (**D**) Several strains encode an additional PL1 pectate lyase within a conserved region, enhancing growth with pectin as sole carbon source (purple: strain 83-1 with additional PL1; gray: strain HOT1A3 without). *p < 0.05.

In addition to plasmids, ecological differentiation also relates to varying abilities for microbial interactions^57^. In this context, strains MIT1002 and EZ55 are naturally associated with *Prochlorococcus* cyanobacteria, to whom they establish mutualistic relationships by alleviating oxidative stress or nutrient limitation during extended periods of darkness^58–61^. Here, we demonstrate additional features that may support their co-existence. Specifically, only MIT1002 harbors a gene cluster encoding the potential for phenol metabolization (Figs. 4A; S2). This ability appears ecologically relevant considering upregulation of phenol hydroxylases in co-culture with *Prochlorococcus* (Table S4 with data from^62^), the common production of phenolics by cyanobacteria^63^, and presence of a homologous gene cluster in *Marinobacter algicola* with comparable association to phototrophs^54^. The *Alteromonas*-*Prochlorococcus* interplay may be further strengthened by metabolic interrelations, as FT-ICR-MS revealed that MIT1002 and EZ55 secrete ecologically relevant exometabolites (Table 1). Secretion of methyl-tryptophan and methyl-indolepyruvate may explain the differential regulation of tryptophan biosynthesis in *Prochlorococcus* when co-cultured with *A. macleodii*^64,65^, especially under restricted photosynthesis^59^. Secretion of asparagine and glutamine (Table 1) indicates exchange of further amino acids, coincident with upregulation of related importers in *Prochlorococcus* when co-cultured^64^. Possible cross-feeding is supported by the potential for mixotrophy^66^ and considerable usage of exogenous amino acids^67^ in environmental *Prochlorococcus* assemblages. Hence, these compounds are possible drivers of varied prokaryotic^68,69^ but also interkingdom interactions, as *A. macleodii* can likewise counteract amino acid deficiency in microalgae^25^.

**Table 1:** Selected exometabolites of *Alteromonas macleodii* strains.

Exact mass (Da)	Predicted compound	Detected in
216.066647	methyl-indolepyruvate	MIT1002
217.098241	methyl-tryptophan	EZ55
131.046208	asparagine	EZ55
145.061869	glutamine	EZ55
198.186345	dodecanamide (fatty acid moiety of 3-oxo-C12-HSL)	MIT1002
296.186680	*N*-3-oxododecanoyl-homoserine lactone (3-oxo-C12-HSL)	HOT1A3
198.113576	*N*-hexanoyl-L-homoserine lactone (C6-HSL)	27126 ^T^

Comparison with prior transcriptomic data^59^ showed that interactions of MIT1002 with *Prochlorococcus* involve several unique genes (Table S4). For instance, differential regulation of unique chemotaxis-, motility- and biofilm-related genes in co-culture may strengthen physical associations^70^ whereas upregulation of a phytase gene might enhance phosphorus acquisition^71^. Overall, the array of interactive features suggests that MIT1002 and EZ55 are adapted to a mutualistic niche with *Prochlorococcus*, a relevant notion considering the cyanobacterium’s reduced metabolic repertoire and importance for biogeochemical cycles^72,73^.

### *Alteromonas*, macroalgae and algal polysaccharides

We herein isolated *A. macleodii* strains BGP6, BGP9 and BGP14 from alginate-supplemented microcosms in the south, equatorial and north Pacific Ocean (Table S1) using analogous procedures that yielded the alginolytic strain *A. macleodii* 83-1 from the Atlantic^33^. Strikingly, the new isolates and strain 83-1 are clonal, featuring only four polymorphisms in 4,801,369 core sites despite being isolated over wide geographic and temporal scales. These observations resemble the isolation of *A. mediterranea* strains with less than 100 polymorphisms from distant locations and years apart^30,38^. In addition, two *A. australica* strains with 99% ANI have been retrieved from opposite global locations^44^, illustrating that highly similar *Alteromonas* spp. are widely distributed over time and space.

The four clonal *A. macleodii* strains encode numerous carbohydrate-active enzymes (CAZymes) and other enzymes involved in carbohydrate-related KEGG categories (Fig. S3A; Table S2), enabling the degradation of various algal polysaccharides^74^ and indicating association with plants^75^. To examine whether these features trigger direct interactions with algae, *A. macleodii* 83-1 was incubated with tissue from the marine macroalga *Ecklonia radiata*, which contains >50% alginate and hence a preferred substrate^33^. However, no significant tissue degradation was observed (Fig. S3B) although epibiotic bacteria cause visible digestion of *Ecklonia* and other macroalgae^76–78^. These observations suggest that *A. macleodii* has limited abilities to attack macroalgal tissue, and potentially utilizes polysaccharide exudates released directly by the macroalga^74^ or by co-metabolizing bacteria^18^. This proposed lifestyle is supported by low *Alteromonas* abundances on wild macroalgae^79^. Alternatively, colonization might occur in a neutral manner, comparable to other *Alteromonas* spp. with a similar CAZyme profile^80^.

Considering nucleotide substitution rates of ca. 10^−8^ per site/year in related *Gammaproteobacteria*^81^, the four clonal strains probably diverged only recently followed by rapid geographic spread, comparable to *Phaeobacter* strains from the same Pacific transect^82^. However, some features illustrate the beginning of differentiation. In BGP9, a 91 Kb region harboring a TonB/ExbBD membrane system and vitamin B_6_ synthesis genes was translocated from chromosome to plasmid (Fig. 2C), which may influence iron and vitamin metabolism^83,84^. The transposed region also harbors the strain’s sole cytochrome *bc*_1_ complex, although essential genes are uncommon on plasmids^85^. At an estimated plasmid loss of ~10^−3^ per cell and generation^86^, this event may pose a considerable risk for survival.

Specific adaptations to algal polysaccharide degradation were also found in strains MIT1002 and AD45, mediated by unique polysaccharide utilization loci (PUL)^87^. Specifically, only MIT1002 harbors a PUL encoding PL22 and PL26 polysaccharide lyases, a GH88 rhamnogalacturonyl hydrolase and several rhamnose-processing genes, allowing growth with rhamnogalacturonan as sole carbon source (Fig. 4B). A PL26-GH88 pair also occurs in the rhamnogalacturonan-degrading flavobacterium *Gramella flava*^88^, indicating co-functionality towards rhamnose-rich polysaccharides. As rhamnogalacturonan is present in widespread marine macroalgae^74^, degradative abilities may strengthen associations between MIT1002 and phototrophs. Homologous PUL in *A. australica* with 80% nucleotide identity (data not shown) demonstrates independent acquisition of these genes by other *Alteromonas* species, comparable to PUL targeting ulvan from green algae^89,90^. Strain AD45 harbors a unique PUL encoding GH85 and GH92 mannosidases and grows with alpha-mannan as sole carbon source (Fig. 4C), but comparable growth of MIT1002 indicates that mannosidase activity also occurs via other encoded GHs (Fig. S3A). Opposed to mannan-degrading marine flavobacteria^91^, strain AD45 does not encode sulfatases and may hence primarily target terrestrial mannans, corresponding to its coastal origin^92^ and the lower degree of sulfatation in terrestrial polysaccharides^93^. A speculative link relates to the isolation of AD45 from the vicinity of aquaculture facilities, where mannan oligosaccharides are increasingly used as feed additive^94^. Overall, presence in diverse terrestrial and aquatic bacteria (Fig. S3C) suggests the PUL as a widespread niche-defining feature.

Finally, we found that adaptation towards algal polysaccharide degradation is also linked to numerical variation in CAZymes, in context of gene dosage effect and substrate affinity^18^. Specifically, *A. macleodii* strains that encode three PL1 pectate lyases grow significantly better on pectin than strains with only two lyases (Figs. 4D; S3A). Enrichment of the third lyase in the exoproteome of strain 83-1^74^ suggests a role in extracellular substrate recognition and initial hydrolysis. Enhanced degradation through higher lyase numbers is consistent with observations in *Zobellia galactanivorans*, a common macroalgal associate and proficient polysaccharide degrader^78^. Overall, the patchy distribution of rhamnogalacturonan, mannan and pectin degradation discriminates *A. macleodii* into specific “polysaccharide utilization types” with distinct ecophysiological roles^95^.

### Cellular communication

**Figure 5:**
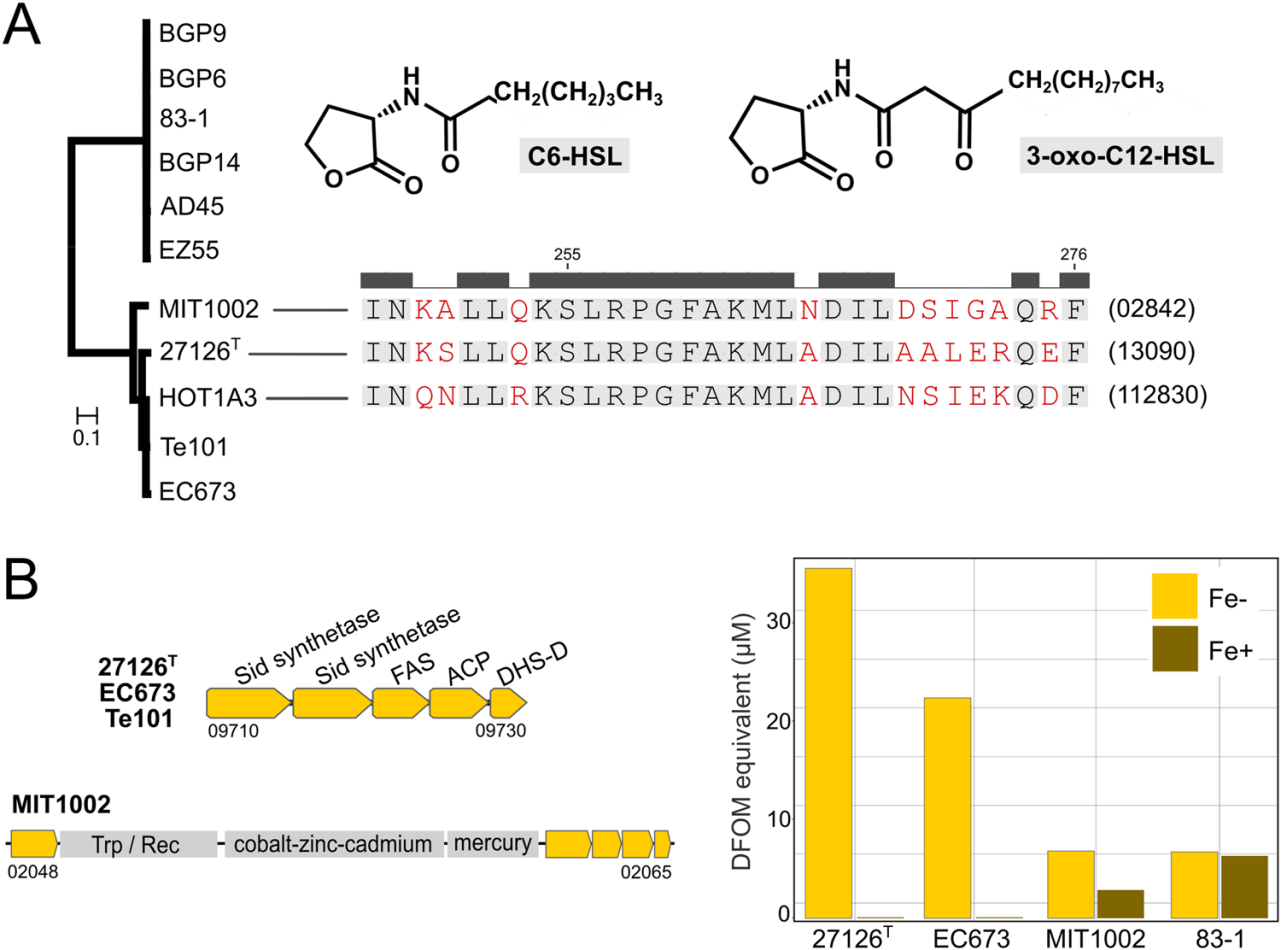
Cellular communication and iron acquisition in *Alteromonas macleodii*. (**A**) Phylogenetic analysis reveals two sequence variants of *N*-acyl amino acid synthase in producers (lower) and non-producers (upper clade) of homoserine lactones (HSL). Accordingly, molecular masses relating to C6-HSL and 3-oxo-C12-HSL were only secreted by strains 27126 ^T^, MIT1002 and HOT1A3 (see Table 1). Strain-specific amino acid substitutions (red) may explain differential HSL production (synthase locus tags in parentheses). (**B**) Gene cluster unique to strains 27126 ^T^, EC673 and Te101 encoding a functional siderophore (locus tags from type strain), with iron-scavenging activity under iron-deplete (Fe−) but not iron-replete (Fe+) conditions in relation to deferoxamine mesylate (DFOM) standard. MIT1002 harbors a nonfunctional cluster after insertion of gene cassettes for cobalt-zinc-cadmium and mercury resistance. Weak signals in MIT1002 and negative control 83-1 under both conditions signify iron-unrelated effects. Sid: siderophore; FAS: fatty acid synthase; ACP: acyl carrier protein; DHS-D: 3-dehydroshikimate dehydratase; Trp: transposase; Rec: recombinase.

Ecological differentiation can also coincide with the potential to coordinate behavior at population level. In this context, we found that *A. macleodii* strains vary in their ability to synthesize homoserine lactones (HSL) for intraspecific communication via quorum sensing^96^. Two gene variants encoding *N*-acyl amino acid synthase occur in *A. macleodii* (Fig. 5A), but masses corresponding to C6-HSL, 3-oxo-C12-HSL and dodecanamide (the fatty acid moiety of 3-oxo-C12-HSL) were only detected in exometabolomes of strains 27126 ^T^, HOT1A3 and MIT1002 (Table 1). The restriction of HSL production to these strains is supported by antismash^97^, which only predicts their sequence variant as functional synthase (Table S1). Accordingly, the autoinducer domain of producers and non-producers has <80% sequence identity (data not shown). Synthase sequences of 27126 ^T^, HOT1A3 and MIT1002 contain different substitutions (Fig. 5A), which potentially explains the observation of HSLs with differing chain lengths^98^. HSLs were only detectable using highly sensitive FT-ICR-MS but not standard bacterial monitor assays^99^, but HSLs can influence chemical interactions and surface attachment even at low concentrations^96,100^. Intraspecific HSL diversity has also been described among symbiotic *Vibrio*^101^, suggesting variable potential for chemical communication as common discriminator of closely related strains.

### Iron acquisition

Successful niche colonization also depends on efficient acquisition of limiting micronutrients, including iron^102^. In this context, only strains 27126 ^T^, EC673 and Te101 harbor a gene cluster for siderophore synthesis with demonstrated iron-scavenging activity (Fig. 5B), likely providing an advantage during iron limitation^103^. The gene cluster is homologous to the petrobactin operon of *Bacillus* spp. (Fig. 3B; 35% amino acid identity) and also occurs in other marine bacteria, suggesting broad ecological relevance^104^. In strain EC673 from the English Channel, the siderophore might support growth with benzoate as sole carbon source (Figs. 3C, S2) by counteracting iron limitation of benzoate breakdown^105^. This scenario could be advantageous considering the anthropogenic input of benzoate in its original habitat^106^. The benzoate cluster is located in a genomic island^32^ and flanked by a transposase, underlining the importance of flexible loci for phenotypic variability. Notably, also *A. mediterranea* EC615 from the English Channel harbors the benzoate-related cluster (Fig. 3C), but encoded on a plasmid^38^. These observations indicate common occurrence and exchange of these genes via mobile genetic elements in habitats where certain chemicals may prevail.

Strain MIT1002 harbors a truncated siderophore cluster, where synthases have been separated by metal-resistance cassettes during the translocation from plasmid to genome (see above). This integration abolished iron-scavenging activity (Fig. 5B), showing that genetic exchange and restructuring of genomic islands can also be disadvantageous.

### Implications for intraspecific interactions and biogeography

**Figure 6:**
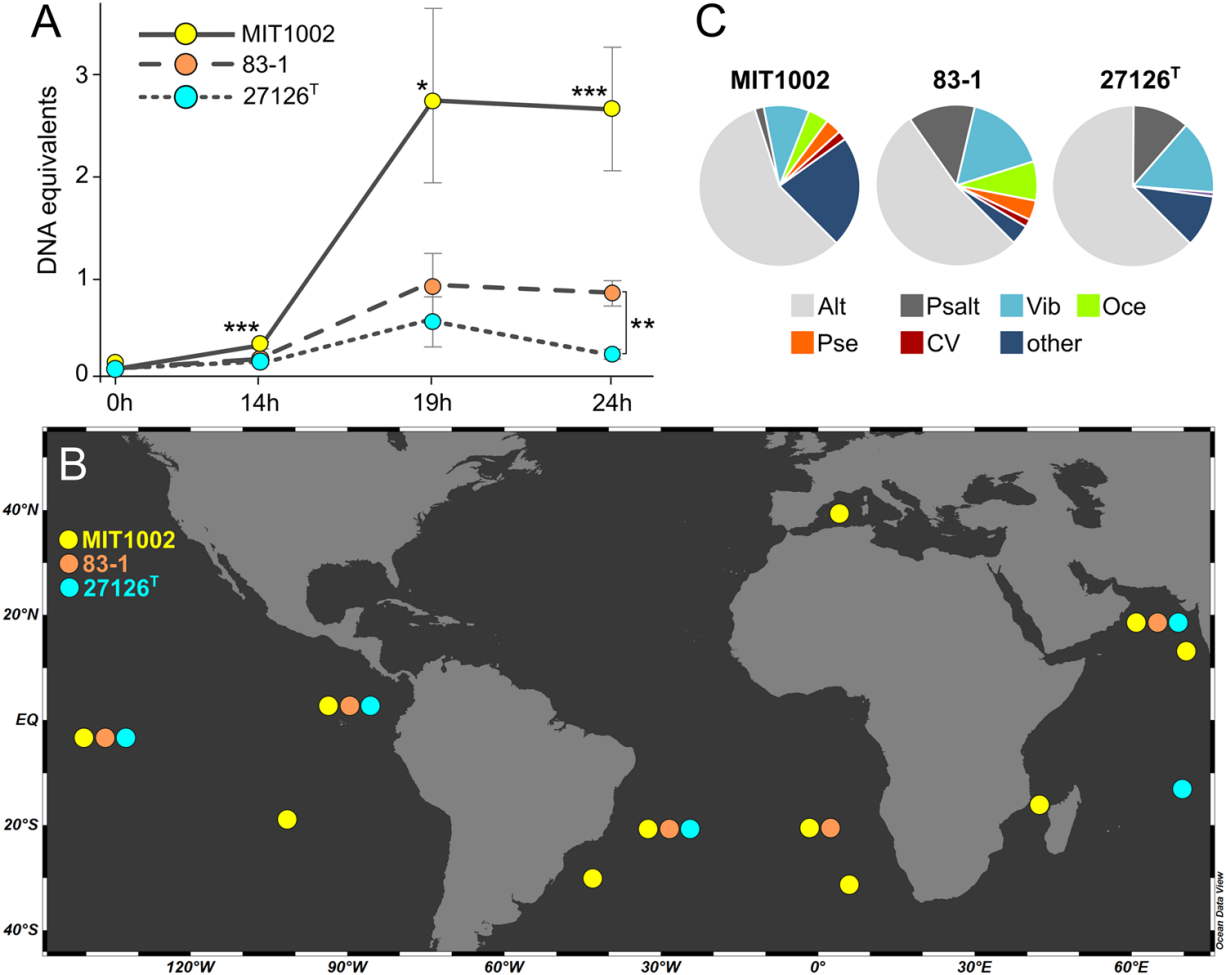
Ecological implications of strain-specific variability in *Alteromonas macleodii*. (**A**) Varying competitiveness of strains MIT1002, 83-1 and 27126 ^T^ in a tripartite co-culture, determined by quantitative PCR of unique genes (*p < 0.01; **p < 0.001; ***p < 0.0001). (**B**) Occurrence of strains MIT1002, 83-1 and 27126 ^T^ in TARA Ocean metagenomes based on BLAST of unique genes (see Table S6 for details). (**C**) Closest relatives of unique genes from strains MIT, 83-1 and 27126 ^T^ based on BLAST against NCBI RefSeq. Alt: *Alteromonadaceae*; Psalt: *Pseudoalteromonadaceae*; Vib: *Vibrionaceae*; Oce: *Oceanospirillaceae*; Pse: *Pseudomonadaceae*; CV: Cellvibrionales (see Table S7 for details).

To address broader eco-evolutionary implications, we asked whether strain-level variability affects population dynamics, competitive abilities and biogeographic distribution^107,108^. For instance, it is known that natural populations of *A. macleodii* can be dominated by specific strains through competitive exclusion^34,109^. To evaluate these aspects, three *A. macleodii* strains with comparable growth in monoculture (Fig. S4) were co-cultured with glucose as sole carbon source, and individual population sizes determined by quantitative PCR of unique genes (Table S5). The tripartite co-culture was dominated by strain MIT1002, which outcompeted both 83-1 and 27126 ^T^ over a period of 24 h (*p* < 0.01). Furthermore, strain 83-1 outcompeted 27126 ^T^ in late exponential phase (*p* < 0.001) (Fig. 6A). Comparable intraspecific differences were also observed in *A. mediterranea*, where greater competitive abilities coincided with higher motility^35^. The putative importance of motility in microbial interactions is supported by upregulation of related genes in MIT1002 when co-cultured with *Prochlorococcus*^62^.

Higher competitiveness of MIT1002 on glucose may provide an advantage in the environment, as glucose is one of the major marine carbohydrates^110^. Accordingly, MIT1002 showed a wider geographic distribution in TARA Ocean metagenomes (Fig. 6B, Table S6), indicating linkages between metabolic abilities and biogeography. Contact with diverse microbiota in different locations may also explain why unique genes of MIT1002 have been acquired from a wider taxonomic range (Fig. 6C; Table S7). These patterns may be amplified by association with *Prochlorococcus*, considering the wide occurrence of the cyanobacterium and higher genetic exchange in host-associated niches^111,112^. In contrast, 27126 ^T^ has been isolated from oligotrophic waters with less biological activity and genetic exchange^113^, and lower growth efficiency on glucose may indicate a *k*-strategist lifestyle. Future co-culturing systems could address how co-existence or competitive exclusion proceed in more complex ecological scenarios, for instance pioneer-scavenger relationships during polysaccharide degradation^18^.

## Conclusions

Here, we extend existing knowledge on (pan)genome evolution and structure in *Alteromonas* by functional perspectives on genome plasticity in twelve *A. macleodii* strains. The shown range of ecological strategies demonstrates that single genospecies can encompass considerable diversity of adaptive features, underlining the importance of polyphasic studies that link bacterial genotypes and phenotypes^114^. The “ecological microdiversity” among strains with >99% 16S rRNA gene identity should be emphasized in microbial diversity studies, which are only beginning to explore the extent of fine-scale variability in natural communities^36^. Notably, phylogenetic relationships only partially corresponded to ecological similarity, illustrated by the patchy distribution of niche-defining metabolic features. Hence, in line with common recombination and genetic exchange^30^, *A. macleodii* appears to perform constant “pathway sampling” that has not (yet) manifested in divergence of specific clades. Metabolic versatility probably facilitates flexible responses to environmental conditions, contributing to the feast-and-famine lifestyle and widespread occurrence of this marine bacterium^24,30^. Sequencing of additional genomes may reveal whether strain-specific abilities translate to the existence of phylogenetic clades with distinct ecological boundaries, corresponding to larger eco-evolutionary concepts^1,115,116^. Our functional-ecological interpretation of the *A. macleodii* pangenome, illustrating the extent of eco-genomic differentiation within bacterial species, has broader implications for niche specialization, microbial interactions and biochemical roles of marine bacteria.

## Materials and Methods

### Isolation and sequencing of *Alteromonas macleodii* strains

Strains BGP6, BGP9 and BGP14 were isolated from alginate-enriched seawater from the south, equatorial and north Pacific Ocean on expedition SO248 with RV *Sonne*^117^. The genomes of BGP strains, MIT1002 and EZ55 were sequenced *de novo* using PacBio II technology (Supplementary Methods). In addition, a number of published closed genomes were analyzed (Table S1).

### Pangenomic and phylogenetic analyses

Core, accessory and unique genes (Table S2) were identified using anvi’o v5.2^118^ following the pangenome workflow of Delmont and coworkers^23^ with minbit parameter 0.5, MCL inflation parameter 10, Euclidean distance and Ward linkage, and NCBI-BLASTp for sequence similarity analysis (see Supplementary Methods for details). For phylogenetic analysis, 92 single-copy core genes (https://help.ezbiocloud.net/ubcg-gene-set) were identified, extracted and aligned using the UBCG pipeline^119^ with *Alteromonas stellipolaris* LMG21861^T^ as outgroup. The alignment was manually checked and submitted to W-IQ-TREE^120^ for calculating a maximum-likelihood phylogeny with 1000 bootstrap replicates and the GTR + G model determined by jModeltest 2^121^. Average nucleotide identities, polymorphic sites and a 16S rRNA gene similarity matrix were calculated using enveomics^122^, ParSNP/Gingr^123^ and BioEdit^124^, respectively. Biosynthetic gene clusters and prophages were predicted using antiSMASH 4.0^97^ and PHASTER^125^, respectively. CAZymes were predicted using dbCAN2^126^ and abundances visualized using R package pheatmap^127^, only considering HMM hits with e-value <10^−23^ and >80% query coverage. Genes were assigned to KEGG categories using KAAS and GhostKoala^128,129^. Annotations were checked using UniProtKB/Swiss-Prot^130^ and Pfam^131^. Amino acid sequences of homoserine lactone synthases were aligned using MAFFT^132^ followed by maximum-likelihood phylogeny using MEGA7^133^ with 1000 bootstrap replicates and the LG + G model determined by ProtTest3^134^. Statistical analyses were done in R v3.5.2^135^ within RStudio (https://www.rstudio.com). Reported significances refer to Wilcoxon rank-sum tests (*p* < 0.05).

### Exometabolomics

All cultivations were done in triplicate using SWM seawater minimal medium^136^. Each replicate was inoculated at 1% (v/v) with precultures grown in 10 mL SWM + 0.1% glucose for 24 h at 20 °C and 140 rpm (washed twice with sterile SWM and diluted to OD600 of 0.1 before inoculation). For exometabolomics, nine strains were inoculated in 50 mL SWM + 0.1% glucose at 0.5% (v/v) in triplicate. After incubation at 20 °C and 140 rpm until late exponential phase, a 20 mL subsample from each replicate was centrifuged for 20 min at 3500 *g* and 4 °C. In addition, three sterile media blanks were incubated and processed in the same manner. Exometabolites were purified from supernatants using solid phase cartridges^137^ followed by ultrahigh-resolution mass spectrometry^138,139^ on a 15 T Solarix Fourier transform ion cyclotron resonance mass spectrometer (FT-ICR-MS) in negative mode (Supplementary Methods). Only peaks present in two biological replicates were considered, and only if detected in technical duplicates measured per replicate. Furthermore, spectra were calibrated and denoised using strict procedures to ensure that only bacterial metabolites were evaluated (Table S3). Tentative identification of masses was done using databases MetaCyc^140^ and KEGG Compounds via R package KEGGREST^141,142^.

### Degradation of different substrates

Degradation of specific carbon sources was tested in SWM supplemented with phenol (final concentration 0.5 mM), toluene (1 mM), xylene (1 mM), sodium benzoate (2 mM), alpha-mannan (Carbosynth YM63069; 0.1% w/v), rhamnogalacturonan (Megazyme P-RHAM1; 0.1% w/v), or pectin (Fluka 76282; 0.1% w/v). Cultures were inoculated with precultures as described above and evaluated by photometry (OD600) or colony-forming units (log CFU mL^−1^) after plating serial dilutions on marine agar (cultures with aromatics subcultured twice before plating). In addition, strain 83-1 was tested for degradation of macroalgal tissue (Supplementary Methods). Briefly, healthy specimens of the brown macroalga *Ecklonia radiata* were incubated with strain 83-1 for 12 days and tissue degradation evaluated in comparison to a control without bacterial addition (*n* = 15).

### Screening for bioactive secondary metabolites

Siderophore production was tested with sterile-filtered supernatants of overnight cultures in iron-deplete vs. iron-replete minimal medium using a modified CAS assay^143,144^ with 50 µM deferoxamine mesylate (DFOM) and sterile medium as positive and negative controls, respectively. Activity was quantified against a seven-point DFOM standard curve (*R*² = 0.981). Production of HSLs was tested by streaking *Alteromonas* colony mass in parallel to the biosensor strains *Chromobacterium violaceum* CV026 and *Agrobacterium tumefaciens* A136 according to Ravn and coworkers^99^, with *Phaeobacter inhibens* DSM17395 as positive control.

### Co-culture and quantitative PCR of unique genes

Quantitative PCR (qPCR) was performed using a LightCycler 480 (Roche, Switzerland) according to Berger and coworkers^145^. For a unique gene of each *A. macleodii* strain, primers were designed using the Roche Universal Probe Library and ordered from TIB MolBiol Germany (Table S5). After confirmation of primer specificity against target and non-target strains, selected strains were grown as mono- and co-cultures in triplicate (inoculated with precultures as described above) in SWM + 0.1% glucose at 100 rpm and 20 °C. DNA was extracted using the Master Pure RNA Purification Kit (Epicentre, Madison, WI) and amplified in 15 µL qPCR reactions (each 10 µL of LightCycler 480 Probes Master, 3 µL PCR-H_2_O, 400 nM of each primer, 200 nM of the respective UPL probe and 5 µL template adjusted to 10 ng µL^−1^). Cycling conditions were 95 °C for 10 min, 45 cycles (95 °C for 10 s, 60 °C for 30 s, 72 °C for 1 s) and 40 °C for 30 s. For each biological replicate, three technical PCR replicates were run. Growth was expressed as DNA equivalents in relation to a five-point DNA standard curve for each strain (*R*^2^ > 0.98).

### Biogeography and taxonomic relatives of unique genes

Three genomic loci specific for strains MIT1002, 83-1 and 27126 ^T^ (Table S6) were searched against TARA Ocean metagenomes using the Sequenceserved-based web application at http://bioinfo.szn.it/tara-blast-server ^146^. Detection was considered positive if at least one gene from two loci was detected with >99% identity and >70% query coverage. Furthermore, unique genes were searched against the NCBI RefSeq Protein database to identify the closest taxonomic relative.

## Supplementary information

Supplementary Information.

Table S1.

Table S2.

Table S3.

Table S4.

Table S5.

Table S6.

Table S7.

## Data Availability

Complete genomes have been deposited at EMBL-EBI under study PRJEB32335 and are also available at IMG^147^ under accession numbers 2738541260, 2738541261, 2738541262, 2738541267 and 2785510739, respectively.
