# Supplementary material for: Genomic, metabolic and phenotypic variability shapes ecological differentiation and intraspecies interactions of Alteromonas macleodii
Source: Sci Rep. 2020 Jan 21;10:809. doi: 10.1038/s41598-020-57526-5 (PMC6972757; doi:10.1038/s41598-020-57526-5)
Supplement: Supplementary file 1 — Supplementary Information. [file 41598_2020_57526_MOESM1_ESM.pdf]

Genomic, metabolic and phenotypic variability shapes ecological differentiation and intraspecies interactions of *Alteromonas macleodii*

Hanna Koch, Nora Germscheid, Heike M. Freese, Beatriz Noriega-Ortega, Dominik Lücking, Martine Berger, Galaxy Qiu, Ezequiel M. Marzinelli, Alexandra H. Campbell, Peter D. Steinberg, Jörg Overmann, Thorsten Dittmar, Meinhard Simon, and Matthias Wietz

SUPPLEMENTARY FIGURES

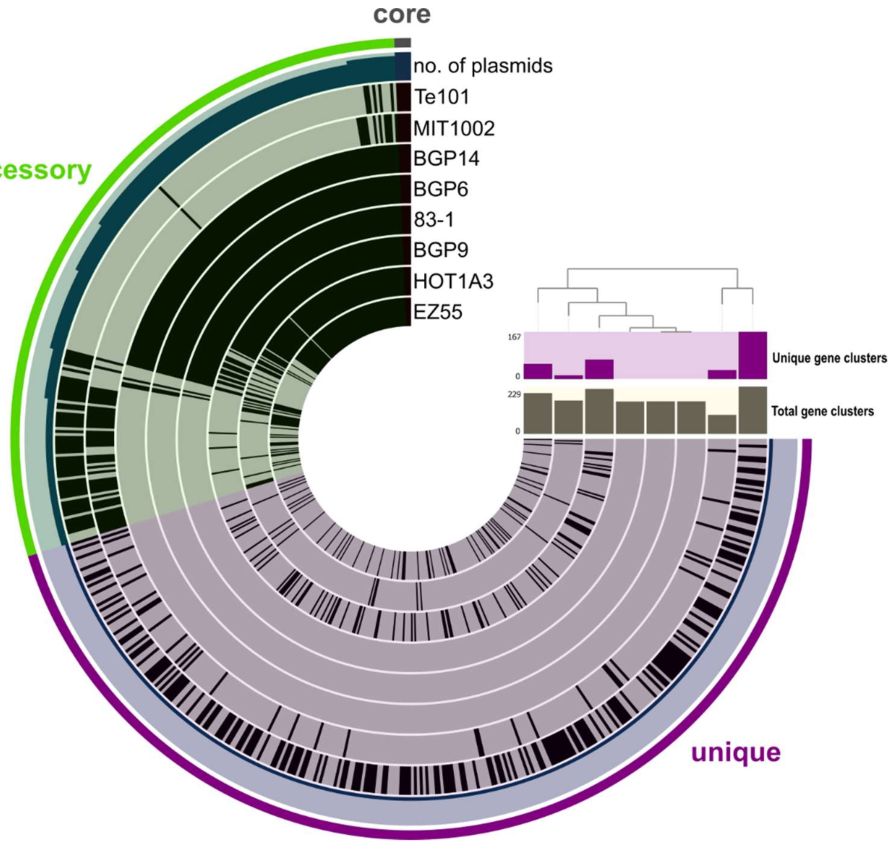

Figure S1 Plasmid structure and content in *Alteromonas macleodii* (presence of genes indicated by black lines).

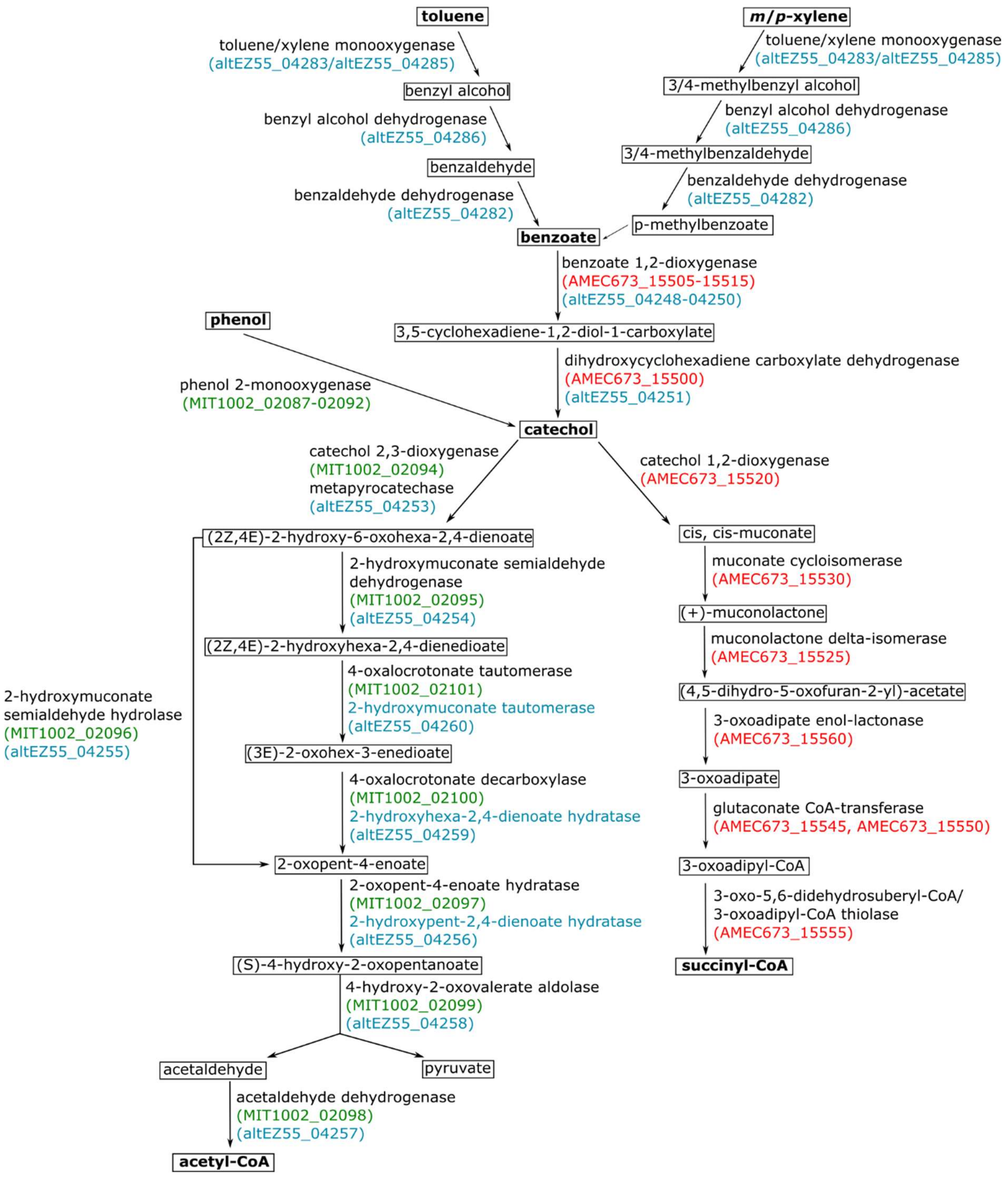

Figure S2 Pathways for degradation of phenol, toluene, xylene and benzoate in *Alteromonas macleodii* EZ55, MIT1002 and EC673, showing locus tags and annotations of involved genes.

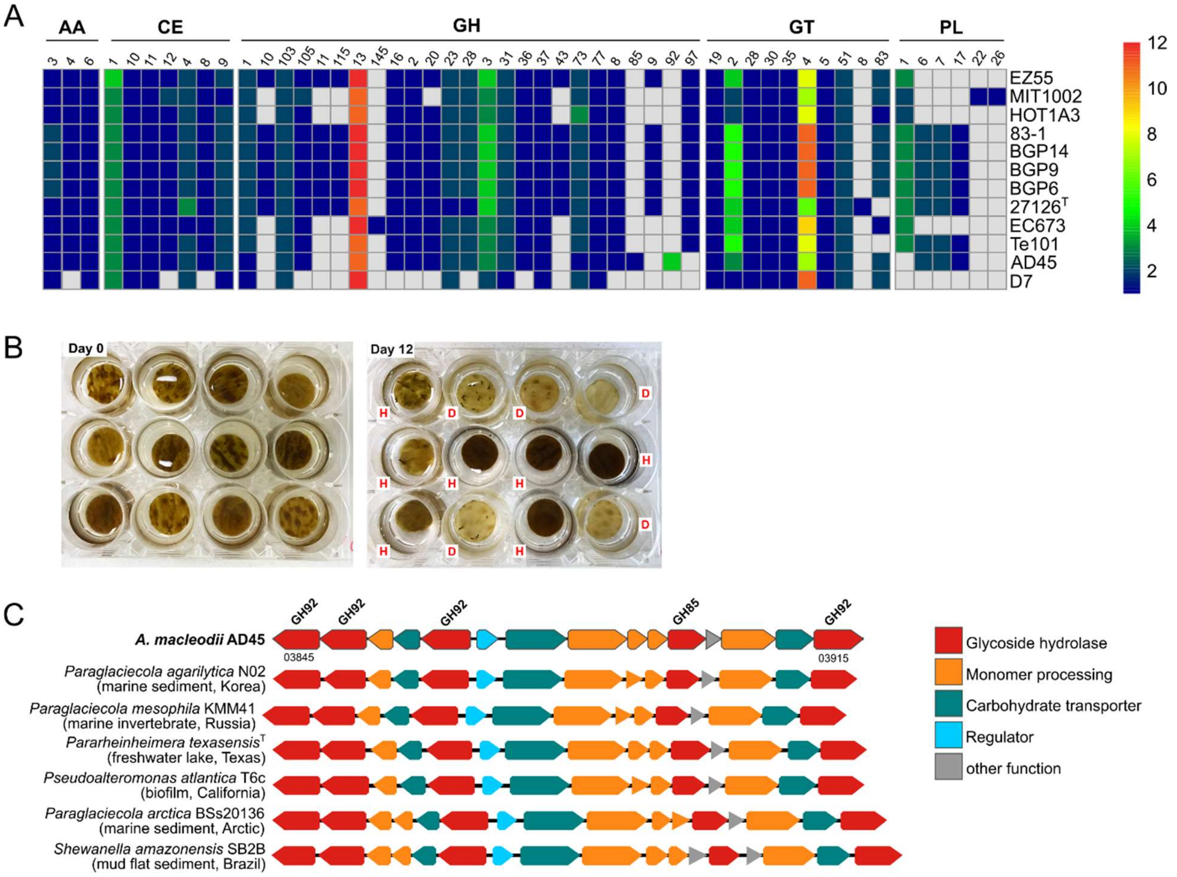

Figure S3 CAZyme diversity and algal tissue degradation in *Alteromonas macleodii*. **A:** Number of genes from different CAZyme families (AA: auxiliary activity; CE: carbohydrate esterase; GH: glycoside hydrolase; GT: glycosyl transferase; PL: polysaccharide lyase). **B:** Tissue from the macroalga *Ecklonia radiata* before/after incubation with *A. macleodii* 83-1 for 12 days, illustrating the appearance of deteriorated (D) and healthy (H) algal tissue. Numbers of deteriorated/healthy tissue pieces after incubation were similar to the control (generalized linear model, $z = 0.43$, $p = 0.67$). **C:** Occurrence of the mannan-related PUL in bacteria from diverse habitats.

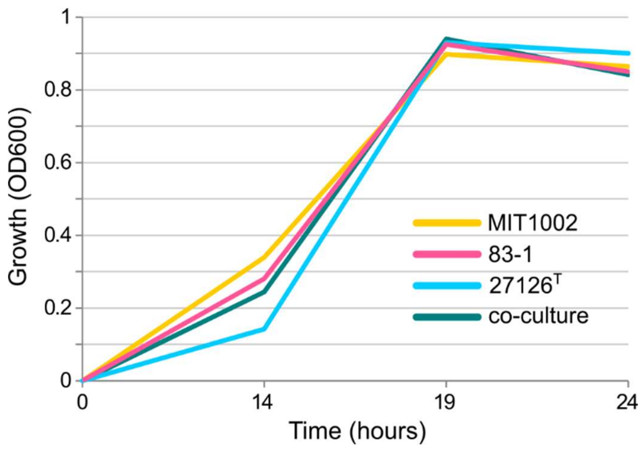

Figure S4 Growth of *Alteromonas macleodii* strains MIT1002, 83-1 and 27126^T in monoculture with glucose as sole carbon source.

SUPPLEMENTARY METHODS

Genome sequencing

Genomic DNA of strains BGP6, BGP9, BGP14, MIT1002 and EZ55 was extracted with the Genomic-tip 100/G kit (Qiagen, Germany). After shearing (g-tubes™; Covaris, Woburn, MA) and monitoring the size range by pulse field gel electrophoresis, DNA fragments were end-repaired and ligated to hairpin adapters using P6 chemistry (Pacific Biosciences, Menlo Park, CA). PacBio reads from SMRT sequencing on a RSII instrument (Pacific Biosciences) were assembled *de novo* using the RS_HGAP_Assembly.3 protocol in the SMRT Portal v2.3. Indel errors were corrected using paired-end Illumina reads from prior sequencing in-house (BGP6, BGP9, BGP14, EZ55) or by collaborating groups (MIT1002)[1] using the Burrows-Wheeler Aligner[2] followed by consensus calling. Each final assembly was circularized and adjusted to the replication system as start point.

Pangenome analyses

The pangenome of twelve *A. macleodii* strains (Table S1) was analyzed using anvi'o v5.2 following the pangenome workflow of Delmont and coworkers[3], also described in detail at http://merenlab.org/2016/11/08/pangenomics-v2. For this, nucleotide sequences were converted into genome databases using tools "anvi-script-FASTA-to-contigs-db" and "anvi-gen-genomes-storage" with default parameters. Annotations of KEGG and COG databases were done following the workflow by Elaina Graham http://merenlab.org/2018/01/17/importing-ghostkoala-annotations and using "anvi-run-ncbi-cogs" with default parameters, respectively. The pangenome database to identify core, accessory and unique genes was generated using the tool "anvi-pan-genome" with minbit parameter 0.5, MCL inflation parameter 10, Euclidean distance and Ward linkage, and NCBI-BLASTp for sequence similarity analysis. For visualization and export of results, the tools "anvi-display-pan" and "anvi-summarize-pan" were used. In addition to bacterial chromosomes, plasmid sequences of eight strains were analyzed without importing the KEGG annotation.

Exometabolomics

Dissolved organic carbon in supernatant filtrates and of solid-phase extracted metabolites was quantified as described previously[4]. Extracts were adjusted to a concentration of 5 ppm carbon for ultrahigh resolution mass spectrometry[5,6] on a 15 T Solarix Fourier transform ion cyclotron resonance mass spectrometer (FT-ICR-MS) coupled to electrospray ionization (Bruker Apollo Daltonics, Bremen, Germany). For each sample, 175 scans in the mass window of 92–1000 Da were accumulated in negative mode. An internal calibration list was generated using Bruker DataAnalysis v4.4 for spectra calibration. Detected mass to charge (m/z) ratios were processed with a customized routine script in Matlab (MathWorks, Natick, MA). Analyte peaks

were separated from instrument noise using the method detection limit[7] and molecular formulae assigned with maximum elemental abundances of $C_nH_nO_nN_4S_1$ (Koch and Dittmar, 2006). Only masses detected in two biological replicates were considered, and only if present in two technical duplicates measured per sample. Masses from procedural blanks or sterile controls were excluded, ensuring that only bacterial metabolites were evaluated (Table S3). As exometabolomes overall correspond to biosynthetic potential[8], masses were tentatively identified using KEGG and MetaCyc databases[9,10]. Few matches among masses >300 Da indicated that some metabolites were modified or condensed during ionization.

Degradation of macroalgal tissue

Healthy adult specimens of the brown macroalga *Ecklonia radiata* were collected from Long Bay (33°57' S, 151°15' E; Sydney, Australia) at 2–4 m depth from sandstone reefs dominated by *E. radiata*, *Sargassum* spp. and various red algae. The healthy phenotype was defined as thalli brown in colour without visible fouling, bleaching or tissue damage. The sampling site was chosen because both healthy and diseased algae occur all year round at Long Bay[11]. Samples were taken from the secondary lamina of algae, placed individually in sealed bags *in situ*, and transferred to the laboratory on ice. After washing with filtered seawater to remove unattached microbes, circular sections (d = 1.8 cm) were cut from the middle section of the lamina using a punch cutter. Only pieces without visible surface damage were selected and aseptically transferred to 12-well plates (n = 15). To each well, *Alteromonas macleodii* 83-1 (precultured to OD600 of 0.5 in Marine Broth 2216 at 24°C and 200 rpm) was added at a final concentration of approx. 5×10^6 cells mL^{-1} (equivalent to bacterial abundance in natural coastal seawater). All plates were incubated at 110 rpm, 22.5°C and a 15:9h light:dark cycle to mimic sublittoral temperature and daylight patterns in summertime Sydney. Every 48h, algal pieces

were checked for deterioration and the supernatant was changed with sterile-filtered seawater. After 12 days, algal pieces were classified as healthy (thallus colour amber/dark brown without obvious bleaching) or deteriorated/bleached (visible whitening). Results were compared to a control incubation (n = 15) prepared in the same manner but without addition of bacteria.

SUPPLEMENTARY TABLES (online only)

Table S1 *Alteromonas macleodii* strains used for pangenome analyses.

Table S2 Core, accessory and unique genes with COG/KEGG classification; average nucleotide identities and 16S rRNA gene similarities of twelve *Alteromonas macleodii* strains.

Table S3 Exometabolites of nine *Alteromonas macleodii* strains as detected by FT-ICR-MS (mean signal intensities of three biological replicates). Individual sheets show (i) all detected molecular masses with formula assignment, (ii) the core metabolome, (iii) the accessory metabolome detected in 2-8 strains, (iv) unique metabolites detected in single strains, and (v) categorization into compound classes. Masses were tentatively identified using KEGG and MetaCyc databases, focusing on masses with 100-300 Da and high signal intensities.

Table S4 Unique genes of *Alteromonas macleodii* MIT1002 differentially regulated in co-culture with *Prochlorococcus* (expression data from Biller and coworkers[62]).

Table S5 Primers for quantitative PCR targeting unique genes of *Alteromonas macleodii*.

Table S6 Detection of *Alteromonas macleodii* strains in TARA Ocean metagenomes based on BLAST of unique genes.

Table S7 Closest sequence relatives of unique genes based on BLAST against NCBI RefSeq.

SUPPLEMENTARY REFERENCES

1 Biller, S. J., Coe, A., Martin-Cuadrado, A.-B. & Chisholm, S. W. Draft genome sequence of *Alteromonas macleodii* strain MIT1002, isolated from an enrichment culture of the marine cyanobacterium *Prochlorococcus*. *Genome Announcements* **3**, e00967--00915, doi:10.1128/genomeA.00967-15 (2015).

2 Li, H. & Durbin, R. Fast and accurate short read alignment with Burrows-Wheeler transform. *Bioinformatics* **25**, 1754-1760, doi:10.1093/bioinformatics/btp324 (2009).

3 Delmont, T. O. & Eren, A. M. Linking pangenomes and metagenomes: the *Prochlorococcus* metapangenome. *PeerJ* **6**, e4320, doi:10.7717/peerj.4320 (2018).

4 Osterholz, H., Niggemann, J., Giebel, H.-A., Simon, M. & Dittmar, T. Inefficient microbial production of refractory dissolved organic matter in the ocean. *Nature Communications* **6**, 7422-7422, doi:10.1038/ncomms8422 (2015).

5 Seidel, M. *et al.* Biogeochemistry of dissolved organic matter in an anoxic intertidal creek bank. *Geochim. Cosmochim. Acta* **140**, 418-434, doi:10.1016/j.gca.2014.05.038 (2014).

6 Osterholz, H. *et al.* Deciphering associations between dissolved organic molecules and bacterial communities in a pelagic marine system. *The ISME Journal* **10**, 1717-1730, doi:10.1038/ismej.2015.231 (2016).

7 Riedel, T. & Dittmar, T. A method detection limit for the analysis of natural organic matter via Fourier Transform Ion Cyclotron Resonance Mass Spectrometry. *Anal. Chem.* **86**, 8376-8382, doi:10.1021/ac501946m (2014).

8 Becker, J. W. *et al.* Closely related phytoplankton species produce similar suites of dissolved organic matter. *Frontiers in Microbiology* **5**, doi:10.3389/fmicb.2014.00111 (2014).

9 Caspi, R. *et al.* The MetaCyc database of metabolic pathways and enzymes and the BioCyc collection of Pathway/Genome Databases. *Nucleic Acids Res.* **42**, D459-471, doi:10.1093/nar/gkt1103 (2014).

10 Kanehisa, M., Furumichi, M., Tanabe, M., Sato, Y. & Morishima, K. KEGG: new perspectives on genomes, pathways, diseases and drugs. *Nucleic Acids Res.* **45**, D353-D361, doi:10.1093/nar/gkw1092 (2017).

11 Marzinelli, E. M. *et al.* Continental-scale variation in seaweed host-associated bacterial communities is a function of host condition, not geography. *Environ. Microbiol.* **17**, 4078-4088, doi:10.1111/1462-2920.12972 (2015).
